# Seroepidemiology of Human Polyomaviruses

**DOI:** 10.1371/journal.ppat.1000363

**Published:** 2009-03-27

**Authors:** Jaime M. Kean, Suchitra Rao, Michael Wang, Robert L. Garcea

**Affiliations:** 1 Department of Microbiology, University of Colorado School of Medicine, Denver, Colorado, United States of America; 2 Department of Pediatrics, University of Colorado School of Medicine, Denver, Colorado, United States of America; 3 Department of Molecular, Cellular, and Developmental Biology, University of Colorado, Boulder, Colorado, United States of America; Brown University, United States of America

## Abstract

In addition to the previously characterized viruses BK and JC, three new human polyomaviruses (Pys) have been recently identified: KIV, WUV, and Merkel Cell Py (MCV). Using an ELISA employing recombinant VP1 capsid proteins, we have determined the seroprevalence of KIV, WUV, and MCV, along with BKV and JCV, and the monkey viruses SV40 and LPV. Soluble VP1 proteins were used to assess crossreactivity between viruses. We found the seroprevalence (+/− 1%) in healthy adult blood donors (1501) was SV40 (9%), BKV (82%), JCV (39%), LPV (15%), KIV (55%), WUV (69%), MCV strain 350 (25%), and MCV strain 339 (42%). Competition assays detected no sero-crossreactivity between the VP1 proteins of LPV or MCV or between WUV and KIV. There was considerable sero-crossreactivity between SV40 and BKV, and to a lesser extent, between SV40 and JCV VP1 proteins. After correcting for crossreactivity, the SV40 seroprevalence was ∼2%. The seroprevalence in children under 21 years of age (n = 721) for all Pys was similar to that of the adult population, suggesting that primary exposure to these viruses likely occurs in childhood.

## Author Summary

Polyomaviruses occupy a replicative niche in animals from birds to humans. Two human polyomaviruses, BKV and JCV, were discovered in 1971 and within the last two years, three new polyomaviruses have been found in humans: KI (KIV), WU (WUV), and Merkel Cell (MCV) polyomavirus. MCV was identified in Merkel Cell carcinomas, a rare skin cancer. To date, it has not been determined what percentage of the human population is exposed to KIV, WUV, and MCV, and when initial exposure to these viruses occurs. We determined that initial exposure to KIV, WUV, and MCV occurs in childhood, similar to that for the known human polyomaviruses BKV and JCV, and that their prevalence is high. We also found evidence that a monkey virus, Lymphotropic Polyomavirus (LPV), likely has a serologically related human counterpart. Another monkey polyomavirus, SV40, was found at ∼2% prevalence, a level that does not support its role in human disease.

## Introduction

Polyomaviruses are small, non-enveloped dsDNA viruses that occupy replicative niches in a variety of vertebrates, and have been extensively studied as oncogenic agents in experimental systems. Five human polyomaviruses have now been identified: BKV [1], JCV [2], KIV [3], WUV [4], and most recently, Merkel Cell Polyomavirus (MCV) [5]. BKV and JCV were discovered in 1971 [1],[2] and are apparently ubiquitous as determined by serology studies, infecting over 80% of some populations by adulthood. Primary infections with BKV and JCV are still not well characterized. Both BKV and JCV persist for life, and their tissue sanctuaries may include mononuclear blood cells (BKV, JCV) and cells of the proximal renal tubule (BKV). Reactivation of these viruses in immunocompromised individuals, results in hemorrhagic cystitis, nephropathy (BKV) and progressive multifocal leukoencephalopathy (JCV).

KIV and WUV were isolated from respiratory specimens by PCR methods, indicating a potential for both disease and transmission via the respiratory route. They have been detected in populations from 4 continents, suggesting a global distribution [6]–[14]. PCR evidence suggests a low prevalence for both KIV and WUV genomes in respiratory samples from individuals [15]. Neither KIV nor WUV DNA has been detected in blood or urine, however, KIV has been detected in fecal specimens [3].

MCV was recently discovered in a Merkel cell carcinoma, integrated into the host cell genome in a manner suggesting a possible relationship to oncogenesis [5]. Interestingly, MCV has a close sequence relationship to the primate Lymphotropic Polyomavirus (LPV). LPV was first isolated in 1979 from an African Green Monkey B-lymphoblastoid cell line [16]. Cellular tropism for LPV includes continuous lines of B lymphoblasts of both human and monkey origin [17]–[19]. Original serologic evidence suggested that an LPV-like virus infection may occur in humans and more recent assays using specific VP1 reagents have suggested that 15–25% of humans are seropositive for LPV VP1-reactive antibodies [20], indicating that exposure to LPV or a virus antigenically related to LPV occurs in the human population. The sequence similarity between LPV and MCV raises the possibility that the seroepidemiology of LPV may be coincident with that of MCV. Another primate virus, SV40, also has been detected in human tissues, but its prevalence and relationship to human disease is controversial.

Little is known about the primary illness associated with infection or potential disease associations of the newly discovered human polyomaviruses. Serologic studies indicate that exposure to BKV and JCV initially occurs during childhood, however, it is unknown when exposure occurs for LPV, KIV, WUV and MCV. In order to study exposure to these viruses in humans, we used recombinant polyomavirus VP1 capsid proteins expressed in *E. coli* in an ELISA assay similar to that described previously for HPV serotype analysis [21],[22]. Our serological results provide data on the prevalence and age-related timeline for infection with the recently discovered polyomaviruses, KIV, WUV, and MCV, as well as for those previously identified: SV40, LPV, BKV, and JCV.

## Results

### Seroprevalence in an adult population

**Figure 1:**
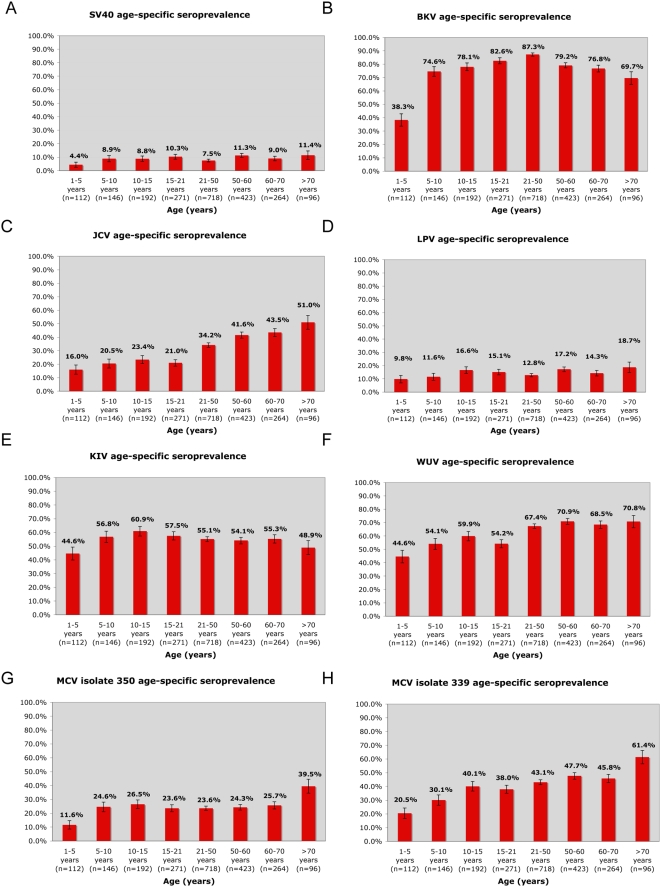
Age-specific seroprevalence detected in a Denver, CO, USA study population (n = 2222) for 7 polyomaviruses known to infect humans. A) SV40 (seroreactivity prior to competition with BKV and JCV VP1 proteins; Figure 3, Table S1); B) BKV; C) JCV; D) LPV; E) KIV; F) WUV; G) MCV isolate 350; H) MCV isolate 339. Standard error bars are shown.

Sera from 1501 adults over the age of 21 were tested for VP1-reactive antibodies using recombinant VP1 capsomeres from SV40, BKV, JCV, LPV, MCV, KIV, and WUV. Seroprevalence values for KIV and WUV were 55% and 69%, respectively. Although seroprevalence values for both KIV and WUV were high in our population, infection with these viruses was not always coincident. We detected differential seroreactivity to 2 different isolates of MCV: 350 (25%) and 339 (46%). Before assessment of crossreactivity, we detected a seroprevalence of 9% for SV40 and 82% for BKV. These values are consistent with previous reports [23],[24]. Thirty-nine percent of the population had JCV VP1-reactive antibodies, and 15% of the population had antibodies against LPV VP1. The LPV seroprevalence was not coincident with the MCV seroprevalence for either isolate, and the respective VP1 proteins did not compete for seroreactivity, as described below. Our data indicate that there may be an age-related waning of BKV VP1 specific antibodies, however, our data do not suggest an age-related waning for any of the other 6 polyomaviruses tested (Figure 1). We did not detect a difference in seroprevalence with respect to gender for any of the 7 polyomaviruses tested (Table 1).

**Table 1:** Seroprevalence of polyomaviruses in adult and pediatric populations in Denver, Colorado, USA stratified by gender and age.

*Age (years)*	*VP1 antigen*															
	SV40		BKV		JCV		LPV		KIV		WUV		MCV isolate 350		MCV isolate 339	
	Male (%)	Female (%)	Male (%)	Female (%)	Male (%)	Female (%)	Male (%)	Female (%)	Male (%)	Female (%)	Male (%)	Female (%)	Male (%)	Female (%)	Male (%)	Female (%)
	(n = 29)	(n = 33)														
1–<3 (n = 62)	1 (3.4)	2 (6.0)	9 (31.0)	9 (27.2)	4 (13.7)	3 (9.0)	3 (10.3)	2 (6.0)	12 (41.3)	8 (24.2)	9 (31.0)	13 (39.3)	1 (3.4)	4 (12.1)	3 (10.3)	8 (24.2)
	(n = 29)	(n = 21)														
3–<5 (n = 50)	1 (3.4)	1 (4.7)	15 (51.7)	10 (47.6)	8 (27.5)	3 (14.2)	4 (13.7)	2 (9.5)	20 (68.9)	10 (47.6)	16 (55.1)	12 (57.1)	6 (20.6)	2 (9.5)	7 (24.1)	5 (23.8)
	(n = 43)	(n = 44)														
5–<8 (n = 87)	4 (9.3)	4 (9.0)	2 (65.1)	33 (75.0)	7 (16.2)	6 (13.6)	4 (9.3)	5 (11.3)	17 (39.5)	27 (61.3)	28 (65.1)	23 (52.2)	8 (18.6)	12 (27.2)	13 (30.2)	12 (27.2)
	(n = 64)	(n = 58)														
8–<12 (n = 122)	5 (7.8)	5 (8.6)	51 (79.6)	45 (77.5)	12 (18.7)	15 (25.8)	9 (14.0)	11 (18.9)	44 (68.7)	32 (55.1)	37 (57.8)	30 (51.7)	12 (18.7)	18 (31.0)	22 (34.3)	18 (31.0)
	(n = 67)	(n = 62)														
12–<15 (n = 129)	7 (10.4)	5 (8.0)	53 (79.0)	49 (79.0)	22 (32.8)	13 (20.9)	12 (17.9)	8 (12.9)	40 (59.7)	40 (64.5)	40 (59.7)	36 (58.0)	16 (23.8)	21 (33.8)	32 (47.7)	24 (38.7)
	(n = 77)	(n = 102)														
15–<18 (n = 179 )	6 (7.7)	13 (12.7)	67 (87.0)	87 (85.2)	12 (15.5)	21 (20.5)	5 (6.4)	17 (16.6)	43 (55.8)	65 (63.7)	37 (48.0)	55 (53.9)	13 (16.8)	25 (24.5)	25 (32.4)	36 (35.2)
	(n = 44)	(n = 48)														
18–<21 (n = 92)	5 (11.3)	4 (8.3)	33 (75.0)	37 (77.0)	12 (27.2)	12 (25.0)	8 (18.1)	11 (22.9)	22 (50.0)	26 (54.1)	25 (56.8)	30 (62.5)	9 (20.4)	17 (35.4)	19 (43.1)	23 (47.9)
	(n = 342)	(n = 376)														
21–<50 (n = 718)	24 (7.0)	30 (7.9)	301 (88.0)	326 (86.7)	124 (36.2)	122 (32.4)	43 (12.5)	49 (13.0)	188 (54.9)	208 (55.3)	234 (68.4)	250 (66.4)	78 (22.8)	92 (24.4)	145 (42.4)	165 (43.8)
	(n = 233)	(n = 190)														
50–<60 (n = 423)	23 (9.8)	25 (13.1)	178 (76.3)	157 (82.6)	86 (36.9)	90 (47.3)	38 (16.3)	35 (18.4)	117 (50.2)	112 (58.9)	171 (73.3)	129 (67.8)	50 (21.4)	53 (27.8)	102 (43.7)	100 (52.6)
	(n = 163)	(n = 101)														
60–<70 (n = 264)	16 (9.8)	8 (7.9)	124 (76.0)	79 (78.2)	69 (42.3)	46 (45.5)	22 (13.5)	16 (15.8)	88 (53.9)	58 (57.4)	115 (70.5)	66 (65.3)	33 (20.2)	35 (34.6)	72 (44.1)	49 (48.5)
	(n = 62)	(n = 34)														
>70 (n = 96)	9 (14.5)	2 (5.8)	42 (67.7)	25 (73.5)	34 (54.8)	15 (44.1)	14 (22.5)	4 (11.7)	26 (41.9)	21 (61.7)	49 (79.0)	19 (55.8)	24 (38.7)	14 (41.1)	39 (62.9)	20 (58.8)

### Age-specific seroprevalence in a pediatric population

The pediatric population consisted of 721 study subjects under the age of 21. As shown in Figure 1, seroprevalence values for these individuals were SV40 (9%), BKV(73%), JCV (21%), LPV (14%), MCV isolate 350 (23%) and MCV isolate 339 (34%), KIV (56%) and WUV (54%). Antibodies to all the VP1 antigens tested were detected in children between 1 and 3 years of age in our study population (Table 1). These data indicate that exposure to all 7 polyomaviruses may occur early in childhood.

### Antibody crossreactivity determined using competition with soluble VP1 proteins

**Figure 2:**
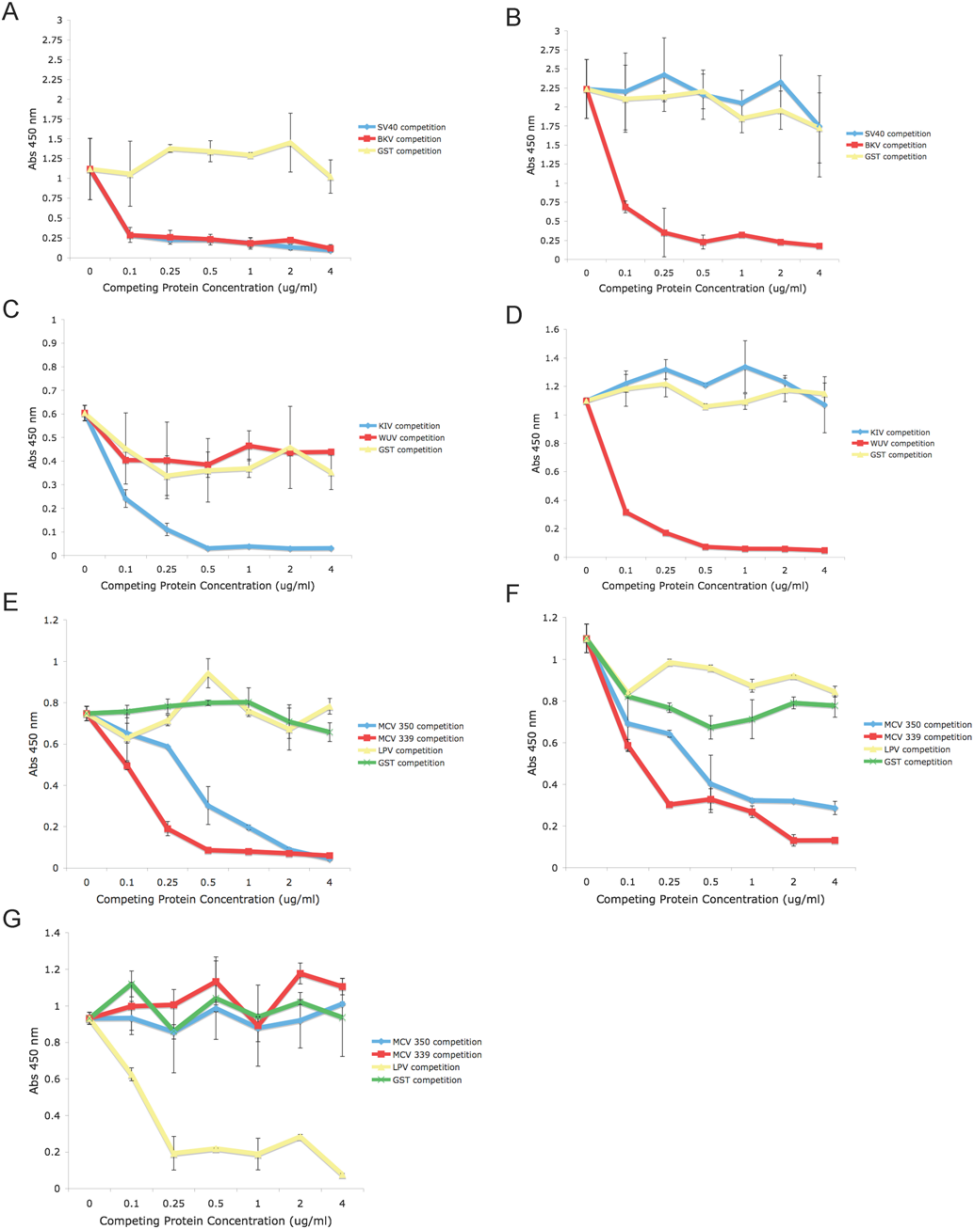
Seroreactivity to VP1 proteins after competition with soluble heterologous VP1 pentamers. A) SV40 seroreactivity competed with BKV; B) BKV seroreactivity competed with SV40; C) KIV seroreactivity competed with WUV; D) WUV seroreactivity competed with KIV; E) MCV isolate 350 seroreactivity competed with MCV isolate 339 and LPV; F) MCV isolate 339 seroreactivity competed with MCV isolate 350 and LPV; and G) LPV seroreactivity competed with MCV isolates 350 and 339. All capsid proteins were competed with themselves as well as with soluble GST as controls.

**Figure 3:**
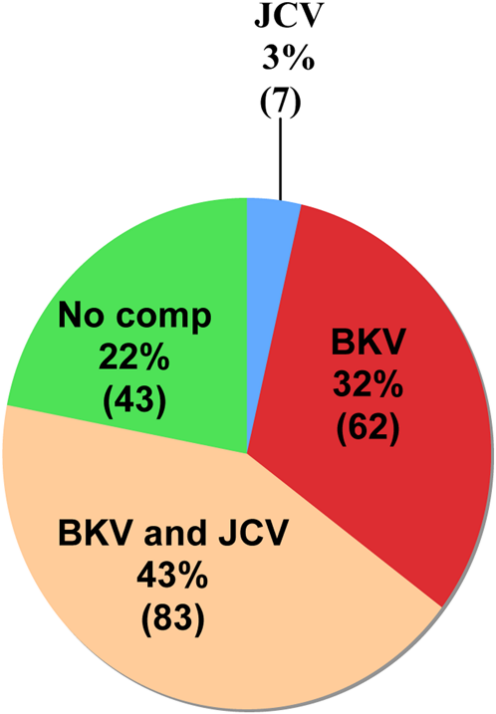
SV40 seroreactive samples competed with JCV and BKV VP1 proteins. 195 samples exhibiting initial SV40 seroreactivity (Figure 1A) were re-analyzed after pre-incubation with soluble BKV and/or JCV VP1. The percentages indicate the number of samples for which SV40 seroreactivity was eliminated by the designated pre-incubation conditions. No competition was observed for 43/195 (23%) of the samples.

Our assay allows testing for possible crossreactivity between VP1 proteins by preincubation of sera with soluble VP1 proteins prior to the specific ELISA antigen. No serologic crossreactivity was detected between the VP1 proteins of LPV and either isolate of MCV or between the VP1 proteins of KIV and WUV (Figure 2). Interestingly, there were 164 sera in our population that were seroreactive to the VP1 protein of MCV isolate 350, but not to 339. Also, 560 samples were seroreactive to isolate 339, but not to isolate 350. Antibody crossreactivity was observed between the VP1 proteins of SV40 and BKV. Therefore, competition assays with soluble BKV and JCV VP1 capsomeres were performed for all SV40 positive samples to determine the extent of SV40 seroreactivity due to crossreactivity with these other viruses. Two hundred samples were found to have SV40 VP1-reactive antibodies, 5 of these samples were not found to have seroreactivity to the VP1 proteins of either BKV or JCV, while 195 samples exhibited coincident seroprevalences with SV40 and BKV and/or JCV (Figure 3). Of the 195 coincident samples, 83 (43%) samples were competed using VP1 pentamers from both BKV and JCV, while 62 (32%) samples were competed using only BKV VP1 pentamers, and 7 (3%) samples were competed using only JCV VP1 pentamers (Figure 3). The ELISA reactivity of 43 (22%) of the 195 coincident samples were not competed with VP1 capsomeres of either BKV and/or JCV. Therefore, a total of 48 samples (2% of the study population) exhibited SV40 “specific” antibodies (Figure 3). Of these 48 samples, 16 (33%) were from individuals less than 21 years of age, 20 (42%) were obtained from individuals between the ages of 21 and 55 years, and 12 (25%) were obtained from individuals over the age of 55 years (Table S1). It was not possible from these de-identified samples to determine if the latter group had received SV40-contaminated polio vaccine.

## Discussion

We have determined the seroprevalence for three recently identified human polyomaviruses (KIV, WUV, and MCV) and confirmed the seroprevalence of two previously known human polyomaviruses (BKV and JCV) and two monkey polyomaviruses (SV40 and LPV) in a human population, using a VP1 capsomere-based ELISA. Our study evaluates both large adult (n = 1501) and pediatric populations (n = 721) to determine the overall prevalence and age of first exposure to these viruses. Additionally, our assay evaluated potential serocrossreactivity occurring between these viruses.

Previous studies of human Py serology have used a variety of assays, including hemaglutination inhibition (HI) [25]–[28] and a virus-like particle (VLP) ELISA-based assay [20], [29]–[31], which may not be directly comparable. The hemaglutination inhibition assay requires either intact virions or VLPs and only evaluates a subset of antibodies. HI may be less sensitive for determining BKV and JCV VP1 seroreactivity, as compared to enzyme immunoassays [32]. Moreover, not all polyomaviruses exhibit hemaglutination (*e.g.*, SV40) and HA has not yet been assessed for KIV, WUV and MCV. A VLP-based ELISA may present conformational epitopes and increased specificity over HI. However, while the VLP-based assay may measure only a subset of antibodies, the capsomere assay has the advantage of measuring all VP1-reactive antibodies, and the use of a casein-glutathione conjugate sterically projects the capsomeres from the well surface, allowing their full exposure to the sera. Nonetheless, seroprevalence determinations are likely somewhat dependent on specific conditions of the assay.

Our observed seropositivity for both WUV (69%) and KIV (55%) was high, despite a low reported detection rate in respiratory tract isolates using PCR [7],[12],[13],[33],[34]. The PCR data likely represent active infection or ongoing co-infection, rather than overall exposure rates. From the age stratification data, it appears that primary infection with these viruses occurs during early childhood, with 35% positive between ages 1–3 for WU and 32% positive for KIV. There was no cross-reactivity between WUV and KIV, which may have been expected given only 65% amino acid identity between their VP1 proteins [4].

We found differential seroreactivity to MCV isolates 350 (25%) and 339 (42%). However, these are not true viral isolates but rather PCR amplified sequences, since no infectious virus has yet been characterized for any of the new human polyomaviruses. The PCR amplifications may have detected defective genomes or variants with minor sequence mutations that occurred after viral integration (specific mutational events have been reported for the Merkel large T-antigen protein [35], and VP1 mutations might be anticipated if they affected productive infection). The differential reactivity in our analysis may therefore have resulted from one PCR variant having a “less native” conformation of the recombinant VP1 protein used in the assays (although sufficiently native to generate VP1 pentamers by electron microscopy) or mutated in critical amino acids that affect a specific epitope. Interestingly, there were 164 sera in our population that were seroreactive to the VP1 protein of MCV isolate 350, but not to 339. Also, 560 samples were seroreactive to isolate 339, but not to isolate 350, suggesting that both isolates of MCV may circulate in the human population. It remains to be determined when authentic, infectious isolates are characterized whether there is actual strain variation in serology. However, as discussed below for JCV, strain differences likely will not substantially affect overall seroprevalence data.

**Figure 4:**
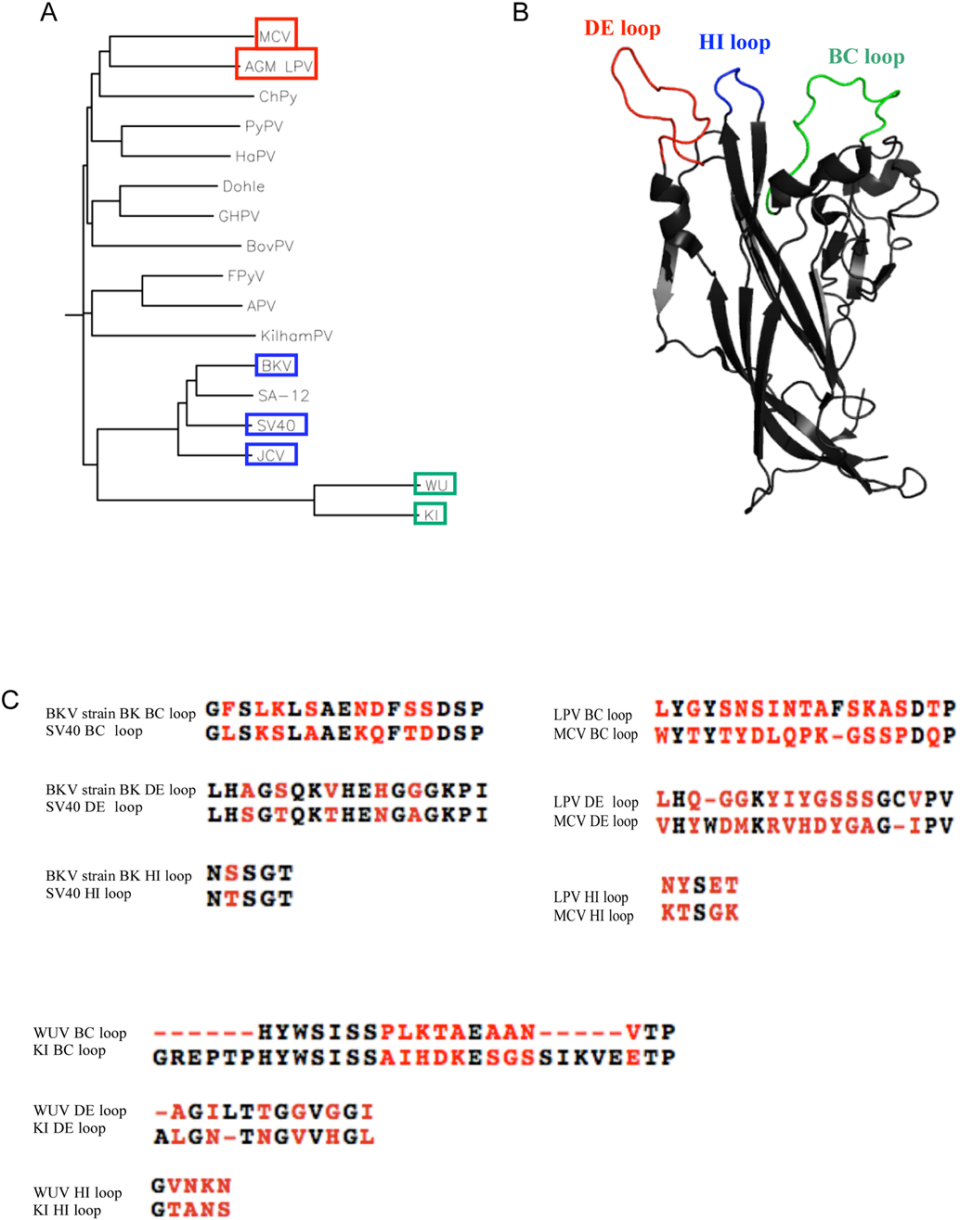
Genetic variability of VP1 proteins among polyomaviruses. A) phylogenetic analysis of 17 known polyomaviruses [45]: B) Crystal structure of VP1 monomer derived from pdb id 3BWQ. PyMOL [46] was used to illustrate the surface variable loop regions C) amino acid primary sequence alignments of VP1 variable loop regions for SV40 and BKV, LPV and MCV, and KIV and WUV.

If these are actually different strains of MCV, the differential prevalence may indicate differential geographic exposure frequencies to these MCV isolates [36],[37]. Also, MCV isolate 339 may contain a sero-dominant MCV VP1 epitope not present in isolate 350. Five amino acid differences occur between the VP1 proteins of MCV 350 and 339. Based on alignment with the VP1 primary amino acid sequence of SV40 and the known structure of SV40 VP1, MCV 339 and 350 do not vary with respect to the surface VP1 exposed variable loop regions thought to comprise the major antigenic determinants, however differences in amino acids occurring close to the surface may indirectly affect loop conformation (Figure S2). Specifically, H288 of MCV 350 is D288 in isolate 339 (Figure S2). Based on alignment with known SV40 VP1 structure, this amino acid difference occurs close to the surface near the HI variable loop. It remains to be determined whether the difference in seroprevalence between MCV isolates 350 and 339 is maintained in suspect disease populations. Our data also support previous studies suggesting that the human population has been exposed to an LPV–like virus, antigenically similar to the primate LPV VP1 [20],[38], and LPV-like sequences have recently been detected in the white blood cells of immunocompromised individuals [39]. Although the recently discovered MCV exhibits a high degree of sequence similarity with LPV (Figure 4) our competition results indicate that MCV and LPV are antigenically distinct viruses.

While the seroprevalence of BKV in our study population is consistent with previous reports [23],[24], the JCV seroprevalence (39%) was somewhat lower [30],[31]. There may be several reasons for this finding: 1) epidemiologic evidence suggests that JCV exposure may differ geographically [24], 2) JCV VP1- specific antibodies may not have as high affinity for the JCV VP1 proteins compared to BKV VP1-specific antibodies, resulting in an overall decreased sensitivity of the JCV assay, 3) although only the MAD-11 JCV genotype has been found to be serologically distinct [40], our assay used only genotype 2B and antibodies other than against genotype 2B may bind that VP1 protein with lower affinity, reducing the sensitivity of the assay.

BKV VP1 IgG antibodies previously have been observed to cross-react with SV40 VP1 [20]. Using recombinant VP1 proteins to compete potential seroreactivity between BKV and SV40, we found the seroprevalence of SV40 to be approximately 2% (Figure 3 and Table S1). In the population of individuals who were seropositive for SV40 and either BKV and/or JCV (n = 195), SV40 seroreactivity could be competed with VP1 pentamers of both BKV and JCV in 43% of the coincident population. SV40 seroreactivity could be competed with BKV VP1 capsomeres alone in 32% of coincident samples, while 3% of coincident samples could be competed only with JCV VP1 capsomeres. If there was no specific seroreactivity we did not assign seropositivity to SV40, since we assume that some antibodies must be specific to SV40 to indicate infection. Indeed, we identified 48 individuals with “specific” SV40 antibodies, supporting our assumption that the SV40 serological response is not restricted only to cross-reactive antibodies with BKV or JCV. It is interesting to speculate that BKV infection might confer protection against SV40, an attribute that may have had a selective advantage at one time. Although we cannot explain the residual SV40 serology of 2%, it is possible that a yet unidentified human polyomaviruses may account for this reactivity.

The external surface variable loop domains of the VP1 proteins may present the dominant epitopes of these viruses. For example, comparison of the loop regions of SV40 and BKV reveals a high degree of similarity. Specifically, the BC loops of SV40 and the BK strain of BKV share 53% identity, while the DE and HI loops exhibit 71% and 80% identity, respectively. Comparison of the loops of LPV and MCV, and those of KIV and WUV, reveals fewer similarities (Figure 4); LPV and MCV exhibit 21% identity in the BC loop, and 26% and 20% in their respective DE and HI loops. KIV and WUV share 33% and 38% respective identity in the BC and DE loops, and only 20% identity in the HI loop. These differences may account for the lack of crossreactivity between these viruses.

Our pediatric population exhibits similar seroprevalence values compared to the adults, indicating that primary infection with polyomaviruses occurs in childhood (Figure 2). We found seropositivity to BKV, WUV, KIV, LPV, and MCV appears in early childhood whereas that for JCV occurs in pre-adolescence (Figure 1). Additionally, our data suggest there may be an age-related waning of BKV VP1 specific antibodies, however, our data do not indicate an age-related waning for any of the other 6 polyomaviruses assayed (Figure 1). While the adult samples are likely representative of the demographics in the Denver area, and reflect healthy individuals within the guidelines for blood donation, the pediatric samples were obtained from inpatients and outpatients at The Children's Hospital Denver and although exclusion criteria were employed, some individuals may have had co-morbid or intercurrent illnesses.

Although animal models have suggested that polyomaviruses could be human tumor viruses [41]–[44] , no substantive cause-effect relationships have yet been established. The debate over SV40 induction of human tumors remains controversial [45], and our data do not support a permissive human infection with SV40, such as seen with the other human polyomaviruses. If SV40 infects humans, it is very limited in scope. Furthermore, if a specific tumor type is presumed attributable to SV40 infection, serological validation now would be an essential factor in the analysis. The discovery of MCV integrated in a specific tumor [5] is a possible indication of the ability of these viruses to contribute to tumorigenesis, but perhaps only in a subset of cell types and only in immunosuppressed individuals. The identification of the human counterpart to LPV may also reveal a connection to human cancer. However, given the high seroprevalence of these viruses, serological support of etiologic connections to specific diseases may be problematic. Nonetheless, these ubiquitous viruses appear to be significant human pathogens in immunosuppressed populations.

## Materials and Methods

### Ethics statement

Plasma samples from healthy adult blood donors were obtained (May and June, 2007) from Bonfils Blood Center (Denver), and pediatric plasma samples were obtained from The Children's Hospital (Denver) using protocols approved by the Colorado Multiple Institutional Review Board. Consent from human participants was not obtained. Samples were de-identified and analyzed anonymously.

### Human plasma samples

To ensure a healthy pediatric population for determination of baseline exposure, the pediatric population excluded patients who had received intravenous immunoglobulin in the preceding year and/or patients who had undergone blood, platelet, or plasma transfusions within two months of obtaining the sample. Children less than one year of age were excluded from the analysis because of possible confounding maternal antibodies.

### Expression plasmids for recombinant GST-VP1 fusion proteins

pGEX4T3 plasmids (GE Healthcare) encoding the VP1 capsid proteins for SV40, LPV, BKV, JCV, KIV,WUV and MCV isolate 339 were obtained and subsequently used for protein production. The MCV isolate 350 *vp1* gene was synthesized according to the published sequence (Genbank accession number NC_010277) (Genscript) and subcloned into the BamHI and XhoI restriction sites of the pGEX4T3 expression vector (GE Healthcare). Subcloning was verified through DNA sequencing analysis of regions flanking the insert using the primers: 5′-GCATGGCCTTTGCAGGGC-3′ and 5′-CGACACTGGCAGAGGCCC-3′.

### Expression and affinity purification of recombinant VP1 proteins

VP1 proteins from SV40, BKV (BK strain), JCV (genotype 2B), LPV, KIV, WUV and MCV were expressed in *E. coli* and purified using affinity chromatography. Briefly, 400 ml cultures of *E. coli* containing the pGEX VP1 expression plasmids were grown at 37°C until OD_600_ = 4.0. Cultures were cooled to room temperature and induced with 1.25 mM IPTG. Cells were then grown at room temperature to an OD_600_ of 8.0. Cells were lysed using a French Press. A clarified lysate was chromatographed using a GSTrap FF affinity column (GE Healthcare) to bind recombinant GST-VP1 capsomeres. Columns were washed with 3 column volumes of Superdex buffer (40 mM HEPES, pH 7.4; 200 mM NaCl; 5% glycerol; 1 mM EDTA, 5 mM DTT, pH 7.2), and GST-VP1 capsomeres were eluted from the column with 10 column volumes of Superdex buffer supplemented with 10 mM reduced glutathione. Protein fractions were combined, buffer exchanged, aliquoted, and stored at −80°C for use in capture ELISA assays.

Soluble VP1 proteins used in competition assays were purified as described above. After buffer exchange, GST-VP1 capsomeres were incubated with glutathione sepharose beads (GE Healthcare) for 1 hr at 4°C. GST tags were cleaved with 50 U of thrombin in 10 ml lysis buffer (40 mM HEPES, pH 7.4; 200 mM NaCl; 5% glycerol; 1 mM EDTA), supplemented with 10 mM DTT. Cleaved VP1 pentamers were collected, concentrated, aliquoted, and stored at −80°C for use in competition assays.

### GST-VP1 capture ELISA

N-terminal GST-tagged pentameric VP1 capsomeres were captured on polysorp 96-well plates (Nunc) using a casein-glutathione conjugate [22]. Plasma samples were diluted 1∶50 in block buffer (5% (w/v) evaporated milk powder; 0.05% (v/v) Tween 20 in phosphate buffered saline, pH 7.4) and incubated with the immobilized GST-VP1 antigen. IgG antibodies were detected using an HRP-labeled secondary antibody, and tetramethylbenzidine with hydrogen peroxide as a substrate. Positive control antibodies were: anti-LPV VP1 monoclonal antibody for LPV VP1 antigen and human sample 637 for both MCV VP1 antigens. We provided BKV VP1 pentamers to Bio-Synthesis Inc, for production of an anti-BKV polyclonal antibody. At a concentration of 1∶50,000, the BKV polyclonal rabbit sera reacted with SV40, BKV, JCV, KIV, and WUV VP1 antigens. Negative controls included capture of GST alone and subsequent incubation with control antibodies for each antigen. ELISA assays for 90 samples were repeated for all 8 antigens tested. Reproducibility was assessed based on agreement of seropositivity for each sample run on different days. Kappa statistics indicated a high degree of agreement between samples: SV40 (0.8515), LPV (0.8218), BKV (0.8668), JCV (0.8062), WUV (0.9076), KIV (0.8851), MCV isolate 350 (0.9446), MCV isolate 339 (0.7976).

### Determination of cut-off-values (COVs)

Net absorbance values were calculated by subtracting the mean absorbance value of the negative control from the mean raw absorbance value read at 450 nm. COVs were determined by ranking net absorbance values and determining the inflection point for each antigen (Figure S1). The COV for the VP1 antigens of SV40, BKV, JCV, LPV, KIV, and MCV isolate 350 VP1 antigens was 0.2. The COV for the VP1 antigen of MCV isolate 339 was 0.22. The COV for WUV VP1 antigen was 0.25.

### Recombinant GST-VP1 capsomere-based competition assay

To determine sera crossreactivity between two or more VP1 proteins, soluble VP1 proteins were purified and plasma samples were pre-adsorbed using these proteins to compete potential crossreactive antibodies in the ELISA assay. Specifically, VP1 capsomere proteins were added to a 1∶50 dilution of plasma and incubated for 1 hr at 4°C prior to incubating samples with immobilized test antigen. Initially, VP1 capsomeres were titrated to determine the concentration needed to effectively compete crossreactive antibodies (Figure 2). From this titration experiment it was determined that 3.0 µg/ml of competing soluble VP1 was sufficient for the competition assays. Competition assays for SV40 seroprevalence were performed on those samples that exhibited coincident seroreactivity for BKV and/or JCV (Figure 3).

### Statistical analysis

Seroprevalence values were rounded to the nearest integer. Chi square and Fischer's exact tests were performed to determine whether there was a difference in seroprevalence for any of the VP1 antigens tested with regard to gender. We stratified the pediatric and adult population by age, as shown in Table 1. Significant differences in cumulative seroprevalence among the age categories were determined by comparing standard errors in each group (Figure 1). SAS version 9.0 was used for all statistical analyses performed.

## Supporting Information

Figure S1 Determination of inflection points for VP1 antigens assayed utilizing the VP1-GST ELISA A) SV40 VP1; B) BKV VP1; C) JCV VP1; D) LPV VP1; E) KIV VP1; F) WUV VP1; G) MCV VP1 isolate 350; H) MCV VP1 isolate 339.
(0.43 MB PDF)

Figure S2 Alignment of primary amino acid sequences for the VP1 proteins of MCV isolate 350 and MCV isolate 339. Variable loop regions are highlighted.
(0.07 MB PDF)

Table S1 Age distribution of SV40 sero-reactive samples after competition with heterologous VP1 capsomeres.
(0.03 MB PDF)
