# Supplementary figures and images for: Seroepidemiology of Human Polyomaviruses
Source: PLoS Pathog. 2009 Mar 27;5(3):e1000363. doi: 10.1371/journal.ppat.1000363 (PMC2655709; doi:10.1371/journal.ppat.1000363)

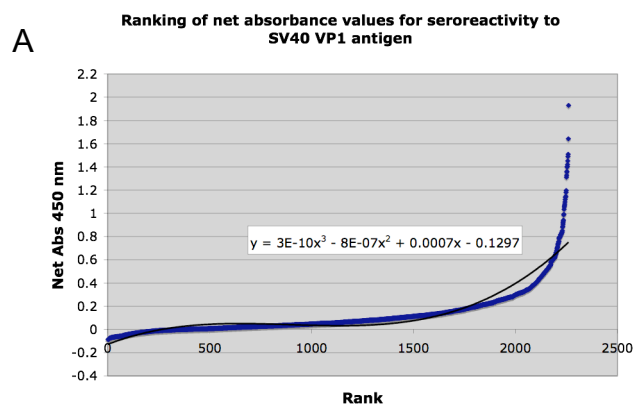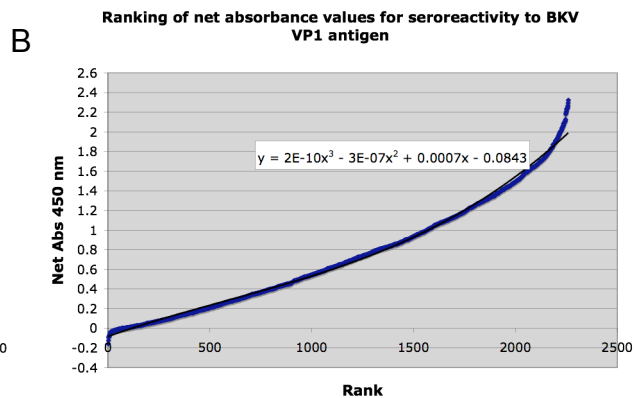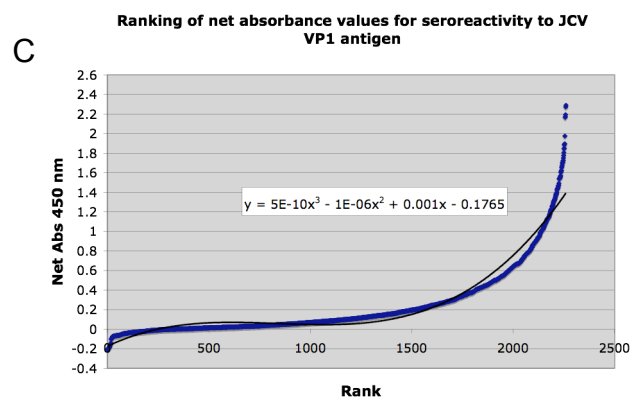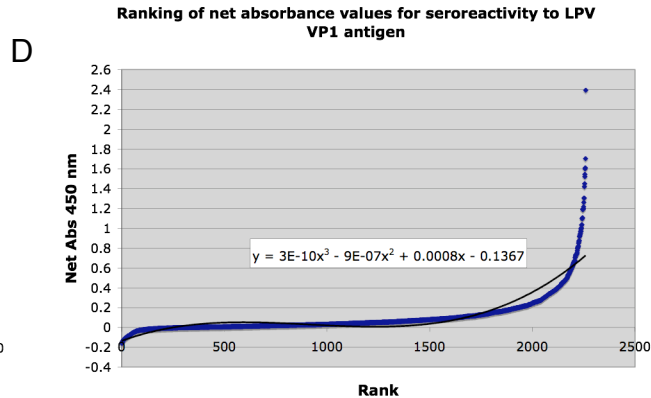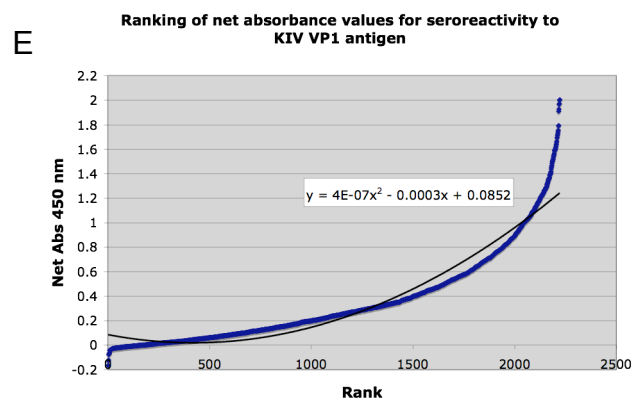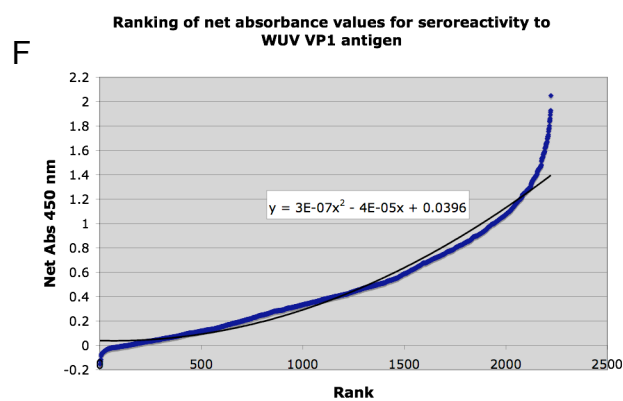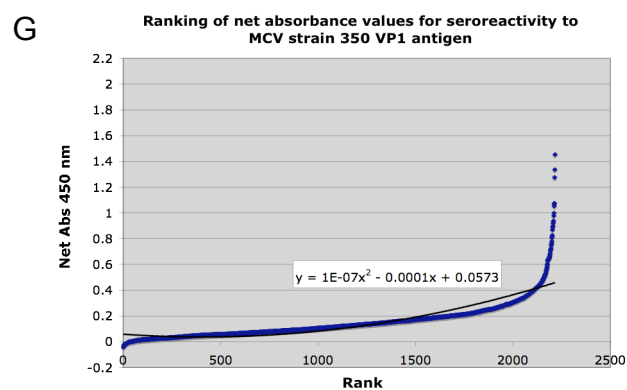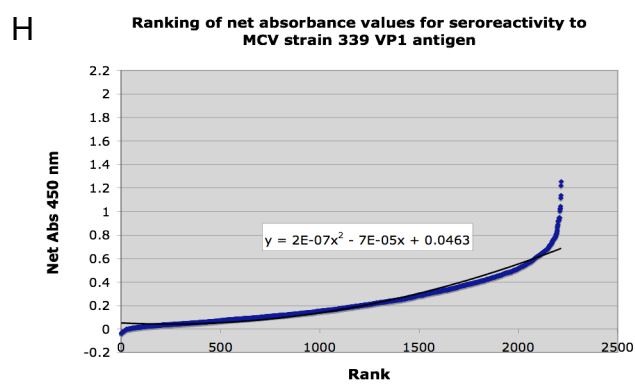

Supplement: Figure S1 — Determination of inflection points for VP1 antigens assayed utilizing the VP1-GST ELISA A) SV40 VP1; B) BKV VP1; C) JCV VP1; D) LPV VP1; E) KIV VP1; F) WUV VP1; G) MCV VP1 isolate 350; H) MCV VP1 isolate 339. (0.43 MB PDF) [file ppat.1000363.s001.pdf]
