# Supplementary material for: Seroepidemiology of Human Polyomaviruses
Source: PLoS Pathog. 2009 Mar 27;5(3):e1000363. doi: 10.1371/journal.ppat.1000363 (PMC2655709; doi:10.1371/journal.ppat.1000363)
Supplement: Figure S2 — Alignment of primary amino acid sequences for the VP1 proteins of MCV isolate 350 and MCV isolate 339. Variable loop regions are highlighted. (0.07 MB PDF) [file ppat.1000363.s002.pdf]

```
                                                   BC loop
MCV_339 MAPKRKASSTCKTPKRQCIPKPGCCPNVASVPKLLVKGGVEVLSVVTGED
MCV_350 MAPKRKASSTCKTPKRQCIPKPGCCPNVASVPKLLVKGGVEVLSVVTGED
        1........10........20........30........40.........

MCV_339 SITQIELYLNPRMGVNSPDLPTTSNWYTYTYDLQPKGSSPDQPIKENLPA
MCV_350 SITQIELYLNPRMGVNSPDLPTTSNWYTYTYDLQPKGSSPDQPIKENLPA
        51.......60........70........80........90.........
                                                   DE loop
MCV_339 YSVARVSLPMLNEDITCDTLQMWEAISVKTEVVGISSLINVHYWDMKRVH
MCV_350 YSVARVSLPMLNEDITCDTLQMWEAISVKTEVVGISSLINVHYWDMKRVH
        101......110.......120.......130.......140........

MCV_339 DYGAGIPVSGVNYHMFAIGGEPLDLQGLVLDYQTQYPKTTNGGPITIETV
MCV_350 DYGAGIPVSGVNYHMFAIGGEPLDLQGLVLDYQTEYPKTTNGGPITIETV
        151......160.......170.......180.......190........

MCV_339 LGRKMTPKNQGLDPQAKAKLDKDGNYPIEVWCPDPSKNENSRYYGSIQTG
MCV_350 LGRKMTPKNQGLDPQAKAKLDKDGNYPIEVWCPDPSKNENSRYYGSIQTG
        201......210.......220.......230.......240........
                                                   HI loop
                                     *
MCV_339 SQTPTVLQFSNTLTTVLLDENGVGPLCKGDGLFISCADIVGFLFKTSGKM
MCV_350 SQTPTVLQFSNTLTTVLLDENGVGPLCKGDGLFISCAHIVGFLFKTSGKM
        251......260.......270.......280.......290........

MCV_339 ALHGLPRYFNVTLRKRWVKNPYPVVNLINSLFSNLMPKVSGQPMEGKDNQ
MCV_350 ALHGLPRYFNVTLRKIWVKNPYPVVNLINSLFSNLMPKVSGQPMEGKDNQ
        301......310.......320.......330.......340........

MCV_339 VEEVRIYEGSEQLPGDPDIVRFLDKFGQEKTVYPKPSVAPAAVTFQSNQQ
MCV_350 VEEVRIYEGSEQLPGNPDIVRFLDKFGQEKTVYPKPSVAPAAVTFQSNQQ
        351......360.......370.......380.......390........

MCV_339 DKGKAPLKGPQKASQKESQTQEL
MCV_350 DKGKAPLKGPQKASQKESQTQQL
        401......410.......420.
```
