# Supplementary material for: Seroepidemiology of Human Polyomaviruses
Source: PLoS Pathog. 2009 Mar 27;5(3):e1000363. doi: 10.1371/journal.ppat.1000363 (PMC2655709; doi:10.1371/journal.ppat.1000363)
Supplement: Table S1 — Age distribution of SV40 sero-reactive samples after competition with heterologous VP1 capsomeres. (0.03 MB PDF) [file ppat.1000363.s003.pdf]

Age (years)	SV40 seroprevalence after competition (n=48)
1- 21 (n=721)	2.2% (16)
21 - 55 (n=938)	2.1% (20)
> 55 (n=563)	2.1% (12)
